# Unusual 4H-phase twinned noble metal nanokites

**DOI:** 10.1038/s41467-019-10764-2

**Published:** 2019-06-28

**Authors:** Wenxin Niu, Jiawei Liu, Jingtao Huang, Bo Chen, Qiyuan He, An-Liang Wang, Qipeng Lu, Ye Chen, Qinbai Yun, Jie Wang, Cuiling Li, Ying Huang, Zhuangchai Lai, Zhanxi Fan, Xue-Jun Wu, Hua Zhang

**Affiliations:** 1 Center for Programmable Materials, School of Materials Science and Engineering, Nanyang Technological University, Singapore, 639798 Singapore; 2 State Key Laboratory of Electroanalytical Chemistry, Changchun Institute of Applied Chemistry, Chinese Academy of Sciences, Changchun, 130022 China; 3 Department of Chemistry, City University of Hong Kong, Kowloon, Hong Kong, China

**Keywords:** Synthesis and processing

## Abstract

Twinning commonly exists in noble metals. In recent years, it has attracted increasing interest as it is powerful to tune the physicochemical properties of metallic nanomaterials. To the best of our knowledge, all the reported twinned noble metal structures exclusively possess the close-packed {111} twinning plane. Here, we report the discovery of non-close-packed twinning planes in our synthesized Au nanokites. By using the bent Au nanoribbons with unique 4H/face-centered cubic*)*/4H crystal-phase heterostructures as templates, Au nanokites with unusual twinned 4H-phase structures have been synthesized, which possess the non-close-packed {10$\bar 1$2} or {10$\bar 1$6} twinning plane. By using the Au nanokites as templates, twinned 4H-phase Au@Ag and Au@PdAg core-shell nanostructures have been synthesized. The discovery of 4H-phase twinned noble metal nanostructures may pave a way for the preparation of metal nanomaterials with unique twinned structures for various promising applications.

## Abstract (web summary)

Twinning is a powerful approach to engineering the physicochemical properties of metallic nanomaterials. Here, the authors discover unusual non-close-packed twinning planes in 4H-phase gold nanokites and show that they can be used as templates to grow 4H-phase twinned nanostructures of other noble metals.

## Introduction

Twinning is commonly observed in noble metal crystals^1–4^. As known, a typical twinned noble metal crystal is composed of two inter-grown sub-crystals, in which one sub-crystal is the mirror image of the other across a plane called the twinning plane^5–7^. In the past two decades, twinning has attracted increasing interest^8–11^, since it can strongly tune the mechanical^12,13^, catalytic^14,15^, and plasmonic^16,17^ properties of metallic nanomaterials. Despite decades of intense research on twinned structures, to the best of our knowledge, all the reported twinned noble metal structures exclusively possess the close-packed {1 1 1} twinning plane^1–4^. Nevertheless, the discovery of non-close-packed twinning in noble metals may expand the scope of twinned noble metal structures and open up more possibilities for engineering the properties of noble metal materials.

Crystal-phase engineering provides a powerful means of tailoring the arrangement of atoms in metal nanocrystals and offers opportunities for the synthesis of unusual metal nanomaterials with tunable physicochemical properties^18,19^. As a typical example, the 4H-phase Au nanoribbons with characteristic stacking sequence of “ABCB” along the close-packed direction^20^ have been used as templates for the epitaxial growth of various metals with unusual 4H phase^21^.

Here, we report the discovery of unusual twinning in our synthesized Au nanokites with non-close-packed twinning planes through crystal-phase engineering. By using the synthesized bent Au nanoribbons with unique 4H/face-centered cubic (fcc)/4H crystal-phase heterostructures as templates, highly anisotropic Au nanokites with non-close-packed twinning planes are discovered. Furthermore, the Au nanokites are used as templates to synthesize the twinned 4H-phase Au@Ag and Au@PdAg core-shell nanostructures.

## Results

### Synthesis of Au nanokites from bent Au nanoribbons

**Fig. 1:**
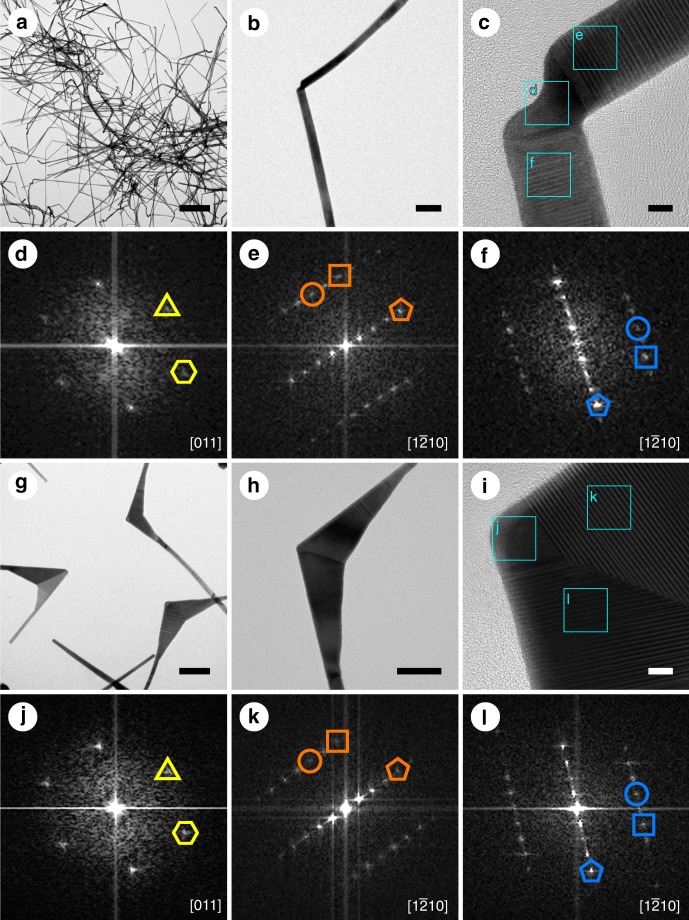
Structural characterizations of bent Au nanoribbons and Au nanokites. **a** Transmission electron microscopy (TEM) image of bent Au nanoribbons. **b** TEM image of a typical bent Au nanoribbon. **c**–**f** High-resolution transmission electron microscopy (HRTEM) image of the curved area of the bent Au nanoribbon with marked areas and their corresponding fast Fourier transform (FFT) patterns. (1 1 $\bar 1$) and (2 0 0) spots of face-centered cubic (fcc) phase are marked with yellow triangles and hexagons, respectively. (0 0 0 4), (1 0 $\bar 1$ 0), and (1 0 $\bar 1$ 2) spots of 4H phase are marked with pentagons, circles, and squares, respectively. The orange and blue represent different 4H sub-crystals. **g** TEM image of Au nanokites. **h** TEM image of a typical Au nanokite. **i**–**l** HRTEM image of the head of the Au nanokite with marked areas and their corresponding FFT patterns. Scale bars, **a** 1 µm; **b** 50 nm; **c** 5 nm; **g** 100 nm; **h** 50 nm; **i** 5 nm

In a typical experiment, unique bent Au nanoribbons (Fig. 1a) were synthesized by using our previously reported synthetic method^22^ with slight modification. The bent Au nanoribbons were characterized by transmission electron microscopy (TEM) and high-resolution TEM (HRTEM) (Fig. 1a–c). HRTEM image and fast Fourier transform (FFT) patterns of a typical bent nanoribbon (Fig. 1c–f) show that it is composed of two pieces of 4H-phase sub-crystals (Fig. 1e, f) connected with a small piece of fcc sub-crystal at the curved area (Fig. 1d), referred to as 4H/fcc/4H crystal-phase heterostructure. The angle of bent Au nanoribbons was measured to be 110.7 ± 23.2° (Supplementary Fig. 1). When it was used as a template for the overgrowth of Au in an *N*, *N*-dimethylformamide (DMF) solution, highly anisotropic Au nanostructures were obtained (Fig. 1g, Supplementary Fig. 2). Atomic force microscopic (AFM) and TEM studies show that most of the obtained Au nanostructures are two-dimensional structures with thickness of about 11.5 nm, possessing one arrow-shaped head and two ribbon-like tails (Supplementary Figs. 3, 4), resembling the shape of a kite and thus being referred to as Au nanokites.

The crystal structure of Au nanokite was characterized by TEM and HRTEM (Fig. 1g–l, Supplementary Fig. 4), showing that it is also composed of two pieces of 4H-phase sub-crystals (Fig. 1k, l) and a small piece of fcc sub-crystal on the tip of its head (Fig. 1j, Supplementary Fig. 5). This result implies that the growth of Au nanokites starts at the 4H/fcc/4H crystal-phase heterostructure of the bent Au nanoribbons. During the growth process, the deposition of reduced Au atoms preferentially occurs in the inner corners of the bent nanoribbons, which are concave surfaces with reentrant grooves. Such reentrant grooves are believed to increase the number of nearest atomic neighbors for the deposition of Au atoms, and thus provide extra stability for Au atoms and promote the preferential growth in the inner corners^23,24^. As the reaction continues, Au atoms prefer to epitaxially grow onto the two 4H-phase sub-crystals of the bent nanoribbon and finally form the two 4H-phase sub-crystals of the Au nanokite. In addition, a slight growth of Au in the fcc sub-crystal at the curved area of the bent nanoribbon was also observed (Fig. 1d, j). It is worth mentioning that there are some random defects distributed in both the bent nanoribbons and nanokites. High-angle annular dark-field scanning transmission electron microscopy (HAADF-STEM) studies show that the stacking faults of 4H phase in both Au nanokites and Au nanoribbons have the “ABCB-CB-ABCB” stacking sequence along the close-packed direction, in which a “CB” planar defect is embedded in the “ABCB” stacking of 4H phase (Supplementary Fig. 6).

### Identification of 4H-phase twinned structures in Au nanokites

**Fig. 2:**
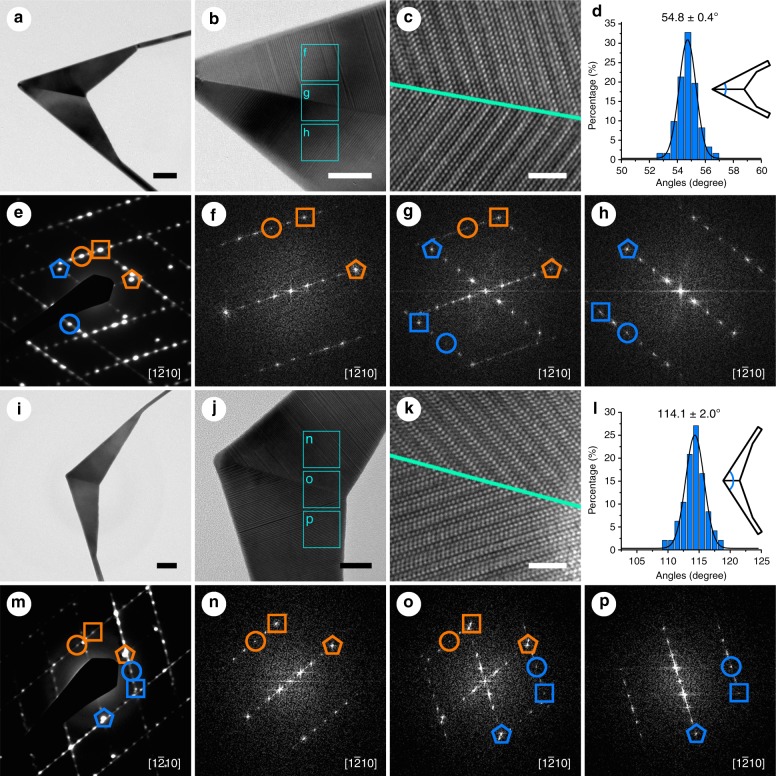
Structural characterizations of acute-angled and obtuse-angled twinned Au nanokites. **a** Transmission electron microscopy (TEM) image of an acute-angled twinned nanokite and **e** the corresponding selected area electron diffraction (SAED) pattern. **b** High-magnification TEM image of the acute-angled twinned Au nanokite with marked areas and **f**–**h** their corresponding fast Fourier transform (FFT) patterns. (0 0 0 4), (1 0 $\bar 1$ 0), and (1 0 $\bar 1$ 2) spots of 4H phase are marked with pentagons, circles, and squares, respectively. The orange and blue represent different 4H sub-crystals. **c** High-resolution TEM (HRTEM) image of the twin boundary of the acute-angled twinned nanokite. The twin boundary is marked in turquoise line. **d** Angle distribution histogram of acute-angled twinned nanokites and a nanokite model showing the measured angle. **i** TEM image of an obtuse-angled twinned nanokite and **m** the corresponding SAED pattern. **j** High-magnification TEM image of the obtuse-angled twinned Au nanokite with marked areas and **n**–**p** their corresponding FFT patterns. **k** HRTEM image of the twin boundary of the obtuse-angled twinned nanokite. The twin boundary is marked in turquoise line. **l** Angle distribution histogram of obtuse-angled twinned nanokites and a nanokite model showing the measured angle. Scale bars, **a**, **i** 50 nm; **b**, **j** 20 nm; **c**, **k** 2 nm

The as-synthesized Au nanokites possess a variety of different angles between two ribbon-like tails (Supplementary Figs. 7, 8). Among nanokites with different angles, two typical structures with well-defined crystal boundaries and angles of 54.4 ± 3.6° (Supplementary Figs. 8, 9) and 111.5 ± 13.4° (Supplementary Figs. 8, 10) were identified. Except them, all the other nanokites give ill-defined crystal boundaries (Supplementary Figs. 7, 11). For the Au nanokites with well-defined crystal boundaries, the two pieces of 4H-phase sub-crystals (Fig. 2a, i) share a straight boundary and appear as mirror image across the boundary (Fig. 2b, j), suggesting the formation of unusual twinned structures of 4H phase. For clarity, hereafter, the twinned nanokites with angles of 54.4 ± 3.6° and 111.5 ± 13.4° are referred to as the acute-angled and obtuse-angled twinned nanokites, respectively.

The crystal structures of twinned Au nanokites were characterized by TEM and selected area electron diffraction (SAED) (Fig. 2). For the acute-angled nanokite (Fig. 2a–h), the SAED pattern (Fig. 2e) can be indexed to 4H-phase Au with zone axis along the [1 $\bar 2$ 1 0] direction, suggesting the top and bottom surfaces of the nanokite are mainly enclosed by {1 $\bar 2$ 1 0} planes^25^. The SAED pattern can be well interpreted by superposition of two sub-crystals of 4H-phase Au along the [1 $\bar 2$ 1 0] direction (Supplementary Fig. 12a). Note that the SAED pattern does not show the diffraction from the fcc sub-crystal at the tip of arrow-shaped head of the Au nanokite because of its small size. To better understand the crystal structure of the acute-angled twinned nanokites, FFT patterns in different areas were obtained (Fig. 2b, f–h), which clearly show two pieces of 4H-phase sub-crystals (Fig. 2f, h). Similar to the SAED pattern, the FFT pattern at the twin boundary shows the superposition of two sub-crystals of 4H-phase Au (Fig. 2g). HRTEM image in Fig. 2c shows the atomic structure of the acute-angled twinned nanokite, with a clear twin boundary marked in turquoise line. Along the twin boundary, two pieces of 4H-phase sub-crystals are mirror images of each other. These results unambiguously uncover an unusual twinned structure of Au nanocrystals. Similar studies show that the obtuse-angled twinned nanokites are also composed of two sub-crystals of 4H-phase Au sharing a twin boundary, representing another form of twinned structures of 4H-phase Au (Fig. 2i–p, Supplementary Fig. 12b). The difference between the acute-angled and obtuse-angled twinned nanokites lies in the orientation of the two 4H-phase sub-crystals. For acute-angled twinned nanokites, one sub-crystal is rotated by 54.8 ± 0.4° with respect to the other sub-crystal (Fig. 2d), while for the obtuse-angled twinned nanokites, the angle of rotation is 114.1 ± 2.0° (Fig. 2l). These results are similar to those obtained by measuring all Au nanokites (Supplementary Figs. 7, 8) and those used for the simulated SAED patterns of the acute-angled and obtuse-angled twinned Au nanokites (Supplementary Fig. 12).

### Theoretical modeling of 4H-phase Au nanokites

**Fig. 3:**
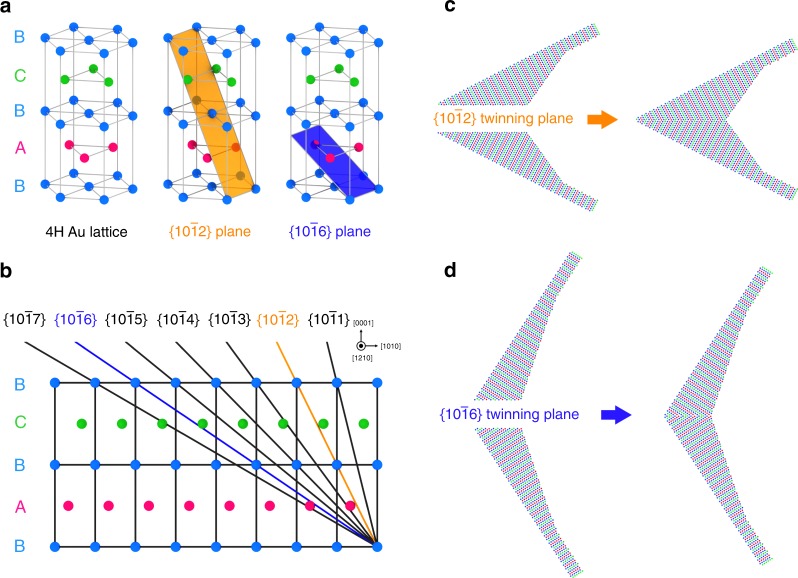
Determination of the atomic structures of 4H-phase twin boundaries in Au nanokites. **a** Schematic models of the hexagonal unit cell of a 4H-phase Au lattice, the {1 0 $\bar 1$ 2} twinning plane, and the {1 0 $\bar 1$ 6} twinning plane. **b** Schematic models of different twinning planes of 4H-phase Au lattice viewed in the [1 $\bar 2$ 1 0] direction. **c**, **d** Schematic models of acute-angled (**c**) and obtuse-angled (**d**) twinned nanokites built from two pieces of 4H-phase sub-crystals with {1 0 $\bar 1$ 2} and {1 0 $\bar 1$ 6} twinning planes

The atomic structures of twin boundaries of Au nanokites were further determined by theoretical modeling. Similar to the twinned 4H-phase Au nanokites, twinning was previously observed in the [1 $\bar 2$1 0] direction for Mg and Mg alloys of hexagonal close-packed (hcp) phase, in which the <$\bar 1$ 0 1 1>{1 0 $\bar 1$ 2} system (where {1 0 $\bar 1$ 2} is the twinning plane and <$\bar 1$ 0 1 1> is the twinning direction) is the dominant twinning mode^26^. By considering the well-established twinning models of hcp Mg, models of the hexagonal unit cell of 4H-phase Au lattice and different twinning planes were built (Fig. 3a, b). Different twinning planes, including {1 0 $\bar 1$ *T*} (*T* = 1, 2, 3, 4, 5, 6, 7), are shown in the [1 $\bar 2$ 1 0] direction (Fig. 3b). Based on these twinning planes, the angles for possible twinned nanokites with different twinning planes were calculated (see the details in Supplementary Methods). The experimental data for acute-angled and obtuse-angled twinned nanokites, that is, 54.8 ± 0.4° (Fig. 2d) and 114.1 ± 2.0° (Fig. 2l), match well with the theoretical data, that is, 54.4° and 114.0°, calculated with the {1 0 $\bar 1$ 2} and {1 0 $\bar 1$ 6} twinning planes, respectively (Supplementary Table 1). Note that 54.4° and 114.0° were used for the simulated SAED patterns of acute-angled and obtuse-angled twinned Au nanokites (Supplementary Fig. 12).

Based on the aforementioned {1 0 $\bar 1$ 2} and {1 0 $\bar 1$ 6} twinning planes, the models of atomic structures of ideal twinned Au nanokites were built from two pieces of 4H sub-crystals (Fig. 3c, d). For the twinned Au nanokite with the {1 0 $\bar 1$ 2} twinning plane, the model was built by seamlessly merging two pieces of 4H-phase sub-crystals with {1 0 $\bar 1$ 2} plane, in which the two 4H-phase sub-crystals share the {1 0 $\bar 1$ 2} twinning plane and are mirror images of each other (Fig. 3c, Supplementary Fig. 13). In a similar process, the model for twinned Au nanokite with the {1 0 $\bar 1$ 6} twinning plane can be built as well (Fig. 3d, Supplementary Fig. 14). The atomic models match well with the corresponding HAADF-STEM images (Supplementary Fig. 15). Further calculation also reveals that the twinning directions for the {1 0 $\bar 1$ 2} and {1 0 $\bar 1$ 6} twinned structures are <$\bar 1$ 0 1 1> and <$\bar 3$ 0 3 1>, respectively (see the details in Supplementary Methods). Therefore, we can conclude that the structures of acute-angled and obtuse-angled twinned nanokites are <$\bar 1$0 1 1>{1 0 $\bar 1$ 2} and <$\bar 3$ 0 3 1>{1 0 $\bar 1$ 6} twinned structures, respectively. To the best of our knowledge, they are previously elusive twinned Au structures with non-close-packed twin boundaries. Structurally, the non-close-packed twin boundaries are distinctly different from the previously observed <1 1 $\bar 2$>{1 1 1} twinned structures of fcc Au, in which the twinning plane is the close-packed {1 1 1} plane^1,4,5^.

### Formation process of twinned Au nanokites

Notably, the formation process of twinned Au nanokites is also different from previous reports, in which twinned structures are formed either during the early stage of nucleation^6,7^ or through the successively developed twinning planes from single-crystalline seeds^8^. Here, the twinned structures of nanokites are formed by using the bent Au nanoribbons with unique 4H/fcc/4H crystal-phase heterostructures as templates. During the growth, the two 4H-phase sub-crystals of the bent Au nanoribbons can spontaneously align their crystal lattice, resulting in the growth of thermodynamically favored twinned structures (Fig. 3c, d) with lower interfacial free energy^27^. Mechanical disturbance such as stirring may be attributed to assisting the angle adjustment, which is similar to that observed in the growth of kinked Au nanowires^28^. Overall, these results illustrate an unusual process for the formation of unique twinned structures.

### Templated growth of other noble metals with 4H-phase twinned structures

**Fig. 4:**
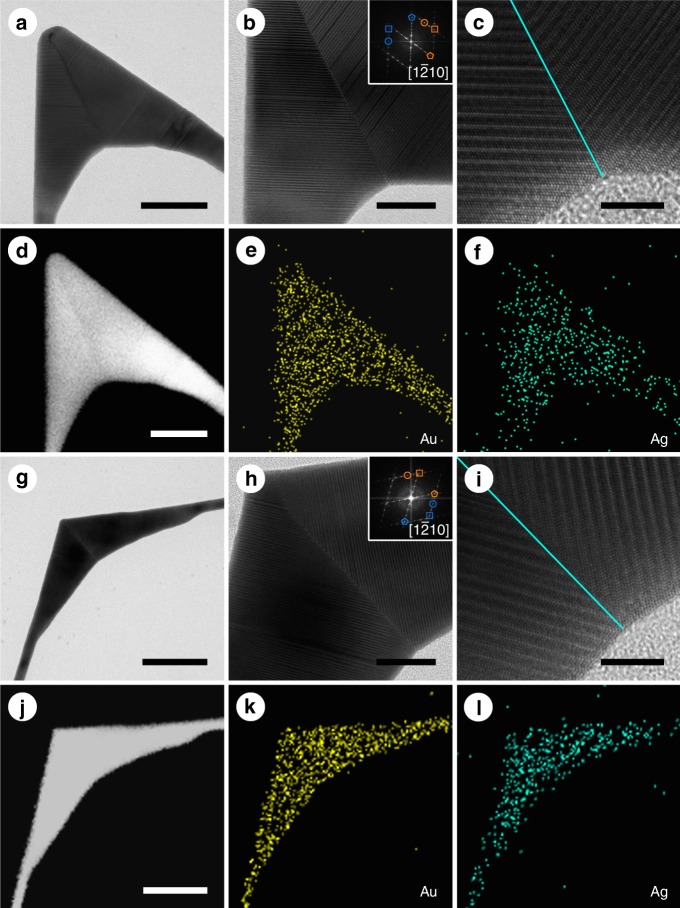
Characterizations of Au@Ag twinned nanokites. **a** Transmission electron microscopy (TEM) image of an acute-angled Au@Ag nanokite. **b** High-magnification TEM image and corresponding fast Fourier transform (FFT) pattern (inset) of the acute-angled Au@Ag nanokite. (0 0 0 4), (1 0 $\bar 1$ 0), and (1 0 $\bar 1$ 2) spots of 4H phase are marked with pentagons, circles, and squares, respectively. The orange and blue represent different 4H sub-crystals. **c** HRTEM image of the twin boundary of the acute-angled Au@Ag twinned nanokite. The twin boundary is marked in turquoise line. **d** Scanning transmission electron microscopy (STEM) and **e**, **f** STEM-energy-dispersive X-ray spectroscopy (STEM-EDX) elemental mapping images of the acute-angled Au@Ag nanokite. **g** TEM image of an obtuse-angled Au@Ag nanokite. **h** High-magnification TEM (HRTEM) image and corresponding FFT pattern (inset) of the obtuse-angled Au@Ag nanokite. **i** HRTEM image of the twin boundary of the obtuse-angled Au@Ag twinned nanokite. The twin boundary is marked in turquoise line. **j** STEM and **k**, **l** STEM-EDX elemental mapping images of the obtuse-angled Au@Ag nanokite. Scale bars, **a**, **d** 50 nm; **b**, **h** 20 nm; **c**, **i** 5 nm; **g**, **j** 100 nm

Impressively, these twinned 4H Au nanokites can be used as templates to grow unusual twinned structures of other noble metals. For example, Au@Ag core-shell twinned nanokites can be synthesized by coating Ag on twinned Au nanokites (Fig. 4). TEM studies (Fig. 4a, b, g, h) show that Ag can epitaxially grow on Au nanokites and leads to the formation of Au@Ag core-shell nanokites with 4H twinned structures, as evidenced by the FFT patterns (the insets of Fig. 4b, h). HRTEM and HAADF-STEM images show that the {1 0 $\bar 1$ 2} and {1 0 $\bar 1$ 6} twinning boundaries of the Au@Ag core-shell nanokites are well preserved after the epitaxial growth process (Fig. 4c, i, Supplementary Fig. 16). STEM images and energy-dispersive X-ray spectroscopy (EDX) elemental mapping results of the Au@Ag nanokites confirm that Ag is homogeneously coated on the Au nanokites (Fig. 4d–f, j–l, Supplementary Fig. 17a, b). Furthermore, Au@PdAg nanokites with well-preserved 4H twinned structures can also be synthesized from the Au@Ag twinned nanokites based on the galvanic reaction between Ag and H_2_PdCl_4_ (Supplementary Figs. 17c, d, 18, 19).

## Discussion

In summary, the selective deposition of Au atoms on the bent Au nanoribbons with 4H/fcc/4H crystal-phase heterostructures is demonstrated and the highly anisotropic Au nanokites are synthesized. Two unusual twinned structures of 4H phase with {1 0 $\bar 1$ 2} and {1 0 $\bar 1$ 6} twinning planes are identified in these Au nanokites, representing previously elusive examples of Au nanocrystals with non-close-packed twinning planes. Furthermore, by using the Au nanokites as templates, the synthesis of other 4H-phase twinned structures, such as Au@Ag and Au@PdAg core-shell nanokites, is achieved. The discovery of unusually twinned noble metal nanostructures will enrich the structural diversity of metal nanomaterials, which might exhibit unique mechanical, catalytic, and plasmonic properties. More importantly, our synthetic method for the formation of twinned nanostructures might offer a strategy for the rational design and synthesis of other unconventional twinned nanomaterials for various promising applications.

## Methods

### Chemicals

Gold(III) chloride trihydrate (HAuCl_4_·3H_2_O), hexane, oleylamine, chloroform, DMF, polyvinylpyrrolidone (PVP, average *M*_w_ ~55,000), silver nitrate (AgNO_3_), palladium(II) chloride (PdCl_2_), hydrochloric acid (HCl), sodium chloride (NaCl), and 1, 2-dichloropropane were purchased from Sigma-Aldrich. All chemicals were used as received without further purification. A 10 mM H_2_PdCl_4_ aqueous solution was prepared by dissolving 17.7 mg PdCl_2_ in 1 mL of 0.2 M HCl solution and further diluting to 10 mL with water.

### Synthesis of bent Au nanoribbons

The bent Au nanoribbons were synthesized according to a previous method^20^ with slight modification. In a typical experiment, 442 mL of hexane, 27.5 mL of oleylamine, and 31.25 mL of 1, 2-dichloropropane were subsequently added into a glass bottle. After thorough mixing, 0.5 g of gold(III) chloride trihydrate was added and dissolved by gentle shaking. The glass bottle was then closely capped and heated for 16 h in an oven at 58 °C. The resulting product was collected by centrifugation (5000 rpm, 3 min), washed with chloroform 4–5 times, and re-dispersed in 14 mL of chloroform, which was then transferred into DMF. Briefly, 1.2 g of PVP was dissolved in 7 mL of chloroform. Then, 7 mL of the Au nanoribbons in chloroform were added under vigorous stirring. After sonication for 20 min, the mixture was incubated overnight in a water bath at 40 °C. The final product was centrifuged (8000 rpm, 3 min), washed twice by DMF solution of 0.1 M PVP, and finally re-dispersed in 7 mL of DMF.

### Synthesis of Au nanokites

PVP (0.8 g) was dissolved in 5 mL of DMF. Then, 27 µL of 0.25 M HAuCl_4_ solution were added. After stirring for 2 min, 5 mL of the bent Au nanoribbons in DMF were added. The mixture was heated in an oil bath at 80 °C for 5 h. The final product was collected by centrifugation (8000 rpm, 3 min), washed with ethanol 3 times, and finally re-dispersed in 2 mL of ethanol.

### Synthesis of Au@Ag nanokites

Prior to the growth of Ag, the as-synthesized Au nanokites were centrifuged and dispersed in 5 mL of DMF. For the synthesis of Au@Ag nanokites, 0.8 g of PVP were dissolved in 5 mL of DMF. Then, 27 µL of 0.25 M AgNO_3_ solution and 5 mL of the Au nanokites in DMF were added. The mixture was heated in an oil bath at 80 °C for 3 h. The final product was collected by centrifugation (8000 rpm, 3 min), washed with ethanol 3 times, and finally re-dispersed in 2 mL of ethanol.

### Synthesis of Au@PdAg nanokites

The synthesis of Au@PdAg nanokites follows a galvanic process. Typically, 0.5 mL of Au@Ag nanokites were added into 4 mL of 10 mM PVP aqueous solution. After sonication, 40 µL of 10 mM H_2_PdCl_4_ solution was added. The mixture was heated in an oil bath at 105 °C for 10 min. The final product was collected by centrifugation (8000 rpm, 3 min), washed with saturated NaCl solution once to remove AgCl and then with ethanol two times, and finally re-dispersed in 0.5 mL of ethanol.

### Characterization

TEM images, HRTEM images, and EDX analysis were acquired using JEOL JEM-2010 and JEM-2100F TEM operating at 200 kV. SAED patterns were acquired using a JEOL JEM-2010 TEM operating at 200 kV. HAADF-STEM images were acquired on a JEOL ARM200F (JEOL, Tokyo, Japan) aberration-corrected TEM operated at 200 kV with a cold field emission gun and double hexapole Cs correctors (CEOS GmbH, Heidelberg, Germany). The convergent semiangle of the probe was set at ~30 mrad. HAADF-STEM images were collected using a half-angle range from about 68 to 280 mrad. AFM images were recorded on a Dimension 5000 AFM (Veeco, CA, USA) under ambient conditions. Scanning electron microscopy images were obtained on a field emission scanning electron microscope (Carl Zeiss Supra 55) operated at 5 kV.

## Supplementary information

Supplementary Information

## Data Availability

The data that support the findings of this study are available from the corresponding author upon reasonable request.
