# Publisher Correction: Unusual 4H-phase twinned noble metal nanokites

**DOI:** 10.1038/s41467-019-11273-y

**Published:** 2019-07-12

**Authors:** Wenxin Niu, Jiawei Liu, Jingtao Huang, Bo Chen, Qiyuan He, An-Liang Wang, Qipeng Lu, Ye Chen, Qinbai Yun, Jie Wang, Cuiling Li, Ying Huang, Zhuangchai Lai, Zhanxi Fan, Xue-Jun Wu, Hua Zhang

**Affiliations:** 1 Center for Programmable Materials, School of Materials Science and Engineering, Nanyang Technological University, Singapore, 639798 Singapore; 2 State Key Laboratory of Electroanalytical Chemistry, Changchun Institute of Applied Chemistry, Chinese Academy of Sciences, Changchun, 130022 China; 3 Department of Chemistry, City University of Hong Kong, Kowloon, Hong Kong, China

**Keywords:** Synthesis and processing

Correction to: *Nature Communications* 10.1038/s41467-019-10764-2, published online 28 June 2019.

The original version of this Article contained an error in the author affiliations.

Affiliation 3 incorrectly read ‘Department of Chemistry, City University of Hong Kong, Kowloon, Hong Kong.’

This has now been corrected in both the PDF and HTML versions of the Article.

