# Supplementary material for: Unusual 4H-phase twinned noble metal nanokites
Source: Nat Commun. 2019 Jun 28;10:2881. doi: 10.1038/s41467-019-10764-2 (PMC6598997; doi:10.1038/s41467-019-10764-2)
Supplement: Supplementary file 1 — Supplementary Information [file 41467_2019_10764_MOESM1_ESM.pdf]

Supplementary Information

Unusual 4H-phase twinned noble metal nanokites

Wenxin Niu[1,2], Jiawei Liu[1], Jingtao Huang[1], Bo Chen[1], Qiyuan He[1], An-Liang Wang[1], Qipeng Lu[1], Ye Chen[1], Qinbai Yun[1], Jie Wang[1], Cuiling Li[1], Ying Huang[1], Zhuangchai Lai[1], Zhanxi Fan[1], Xue-Jun Wu[1], and Hua Zhang[1,3]*

[1]Center for Programmable Materials, School of Materials Science and Engineering, Nanyang Technological University, 50 Nanyang Avenue, Singapore 639798, Singapore.

[2]State Key Laboratory of Electroanalytical Chemistry, Changchun Institute of Applied Chemistry, Chinese Academy of Sciences, Changchun, Jilin 130022, P.R. China.

[3]Department of Chemistry, City University of Hong Kong, Kowloon, Hong Kong, China.

*Corresponding author

E-mail: hzhang@ntu.edu.sg; hua.zhang@cityu.edu.hk

Website: http://www.ntu.edu.sg/home/hzhang/

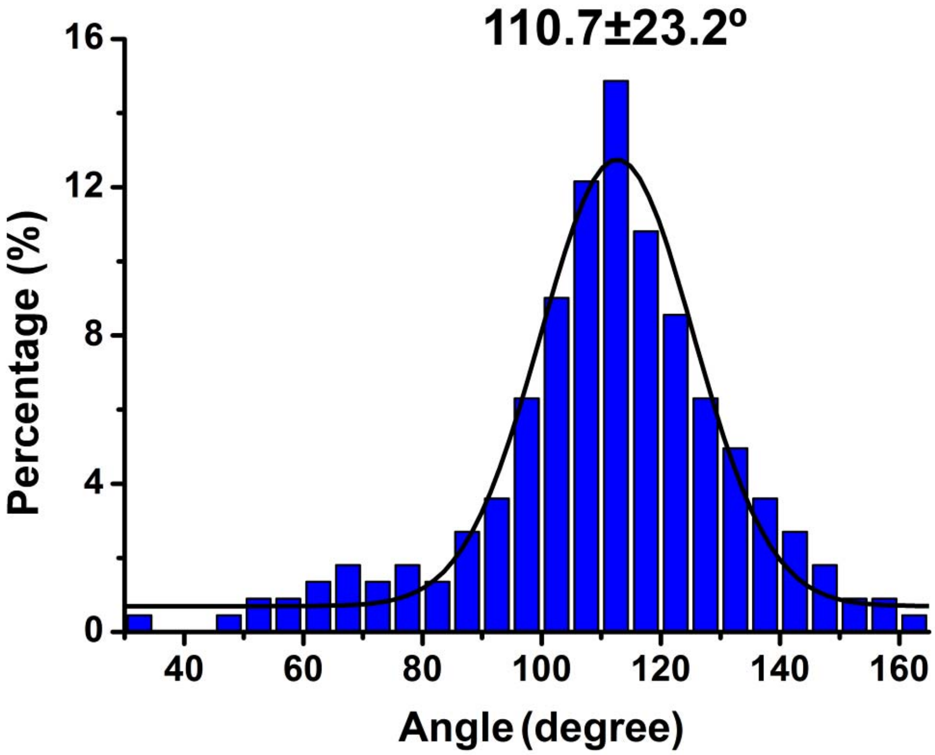

Supplementary Figure 1 | Angle distribution histogram of bent Au nanoribbons.

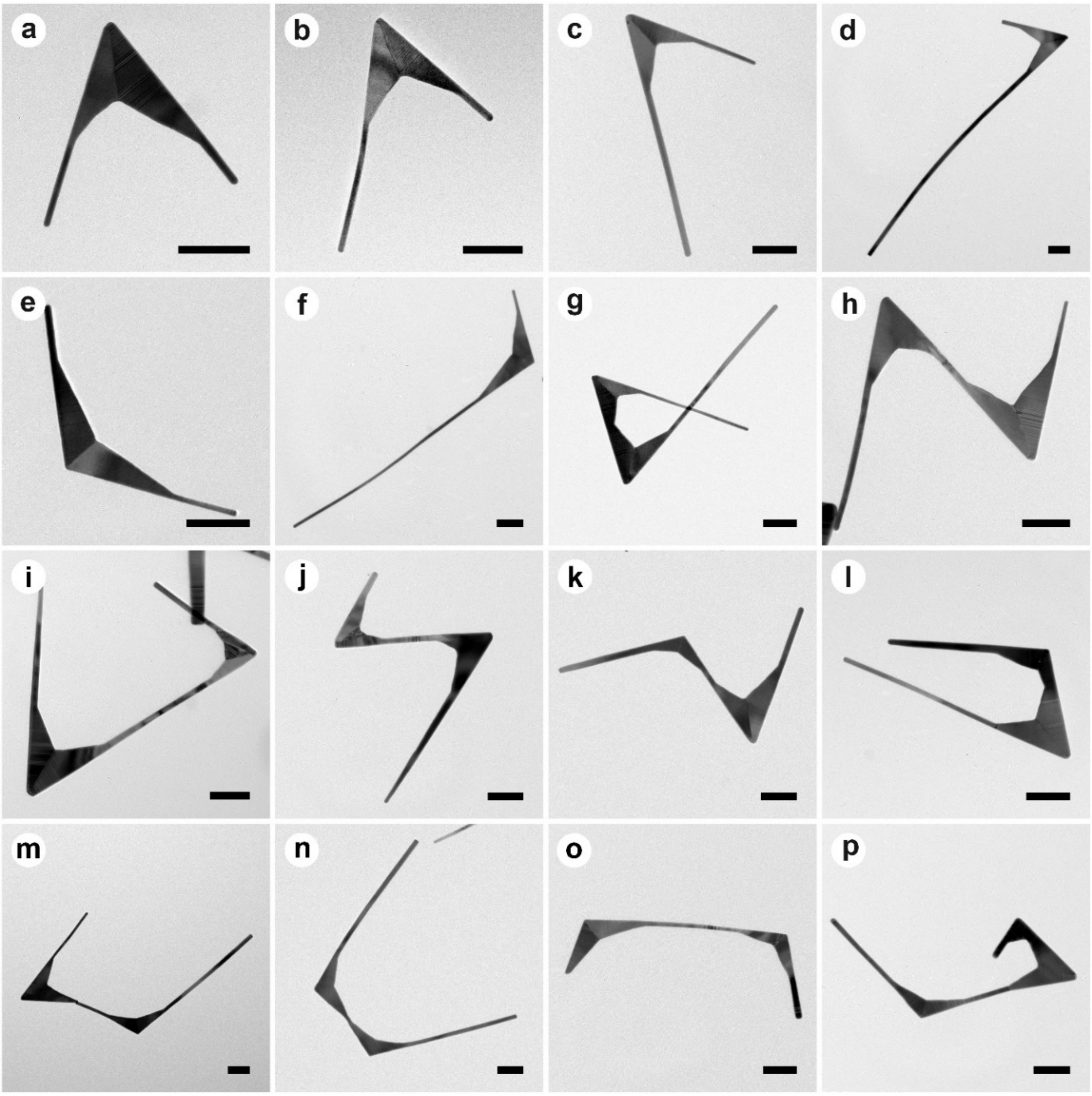

Supplementary Figure 2 | Typical TEM images of Au nanokites. Scale bars, **a-p** 100 nm.

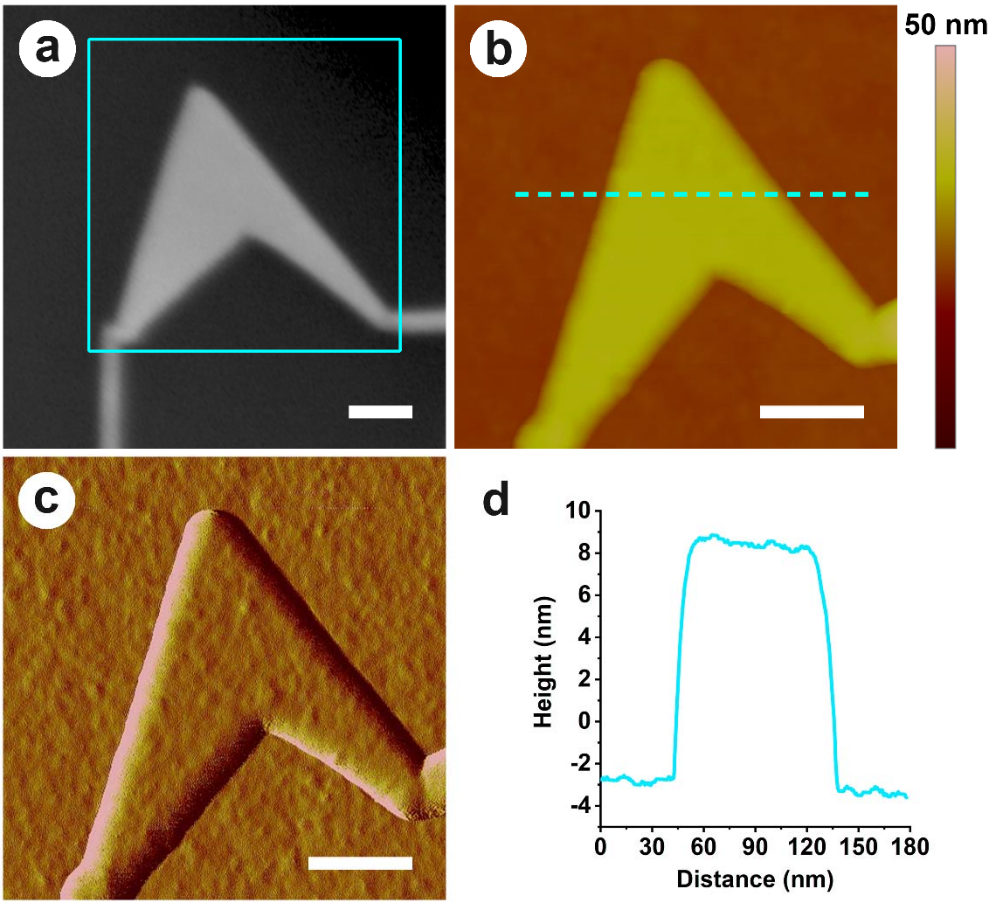

Supplementary Figure 3 | SEM and AFM measurements of a typical Au nanokite. a SEM image, **b** AFM image (height mode), and **c** AFM image (amplitude mode) of the Au nanokite. **d** Height profile of the Au nanokite along the dashed turquoise line in **b**, showing the thickness of about 11.5 nm. Scale bars, **a-c** 50 nm.

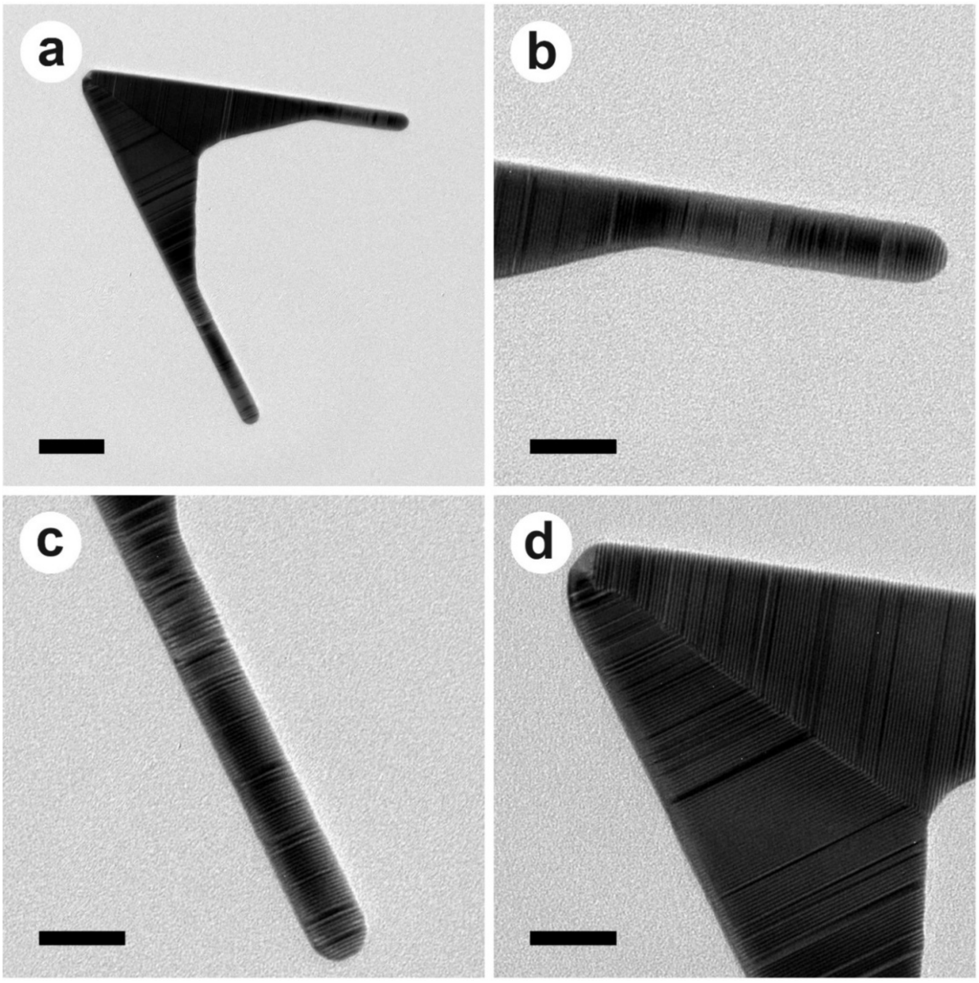

Supplementary Figure 4 | Structures of the head and two tails of an Au nanokite. a TEM image of a single Au nanokite. **b, c** High-magnification TEM images of two 4H-phase tails of the Au nanokite. **d** High-magnification TEM image of the 4H-phase arrow-shaped head of the Au nanokite. Scale bars, **a** 50 nm; **b-d** 20 nm.

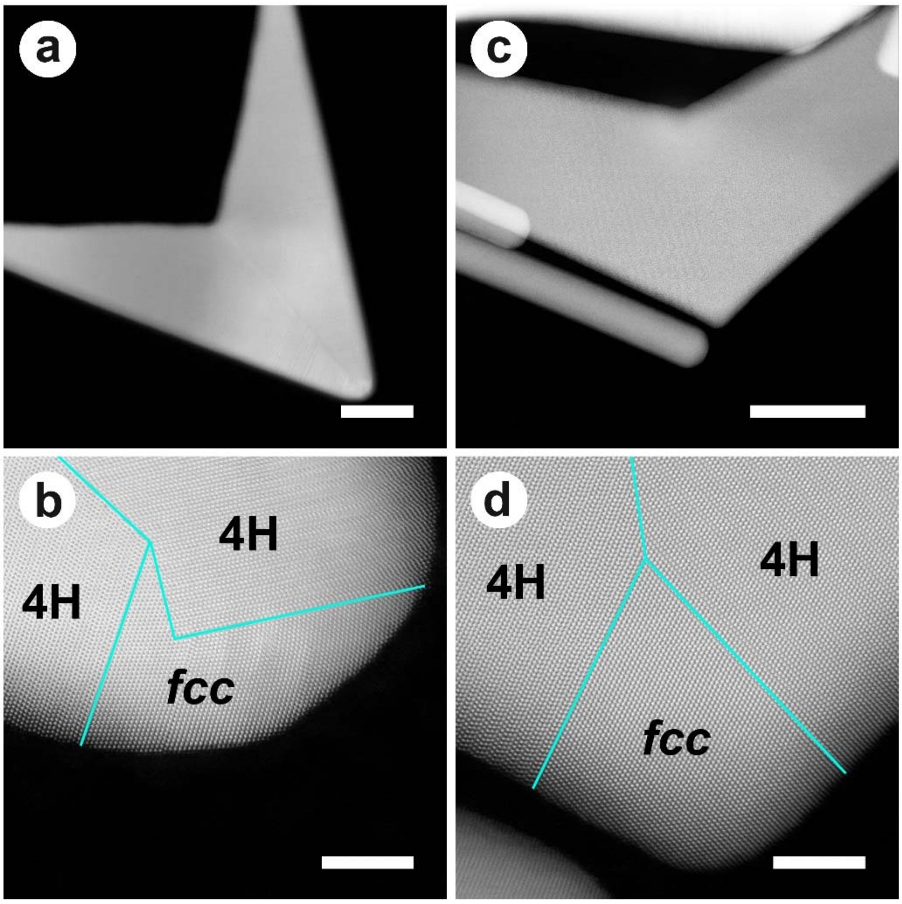

Supplementary Figure 5 | HAADF-STEM images of acute and obtuse Au nanokites. a HAADF-STEM image of an acute-angled Au nanokite. **b** HAADF-STEM image of the tip of Au nanokite in **a**. **c** HAADF-STEM image of an obtuse-angled Au nanokite. **d** HAADF-STEM image of the tip of Au nanokite in **c**. The crystal boundaries are marked in turquoise lines. Scale bars, **a, c** 50 nm; **b, d** 5 nm.

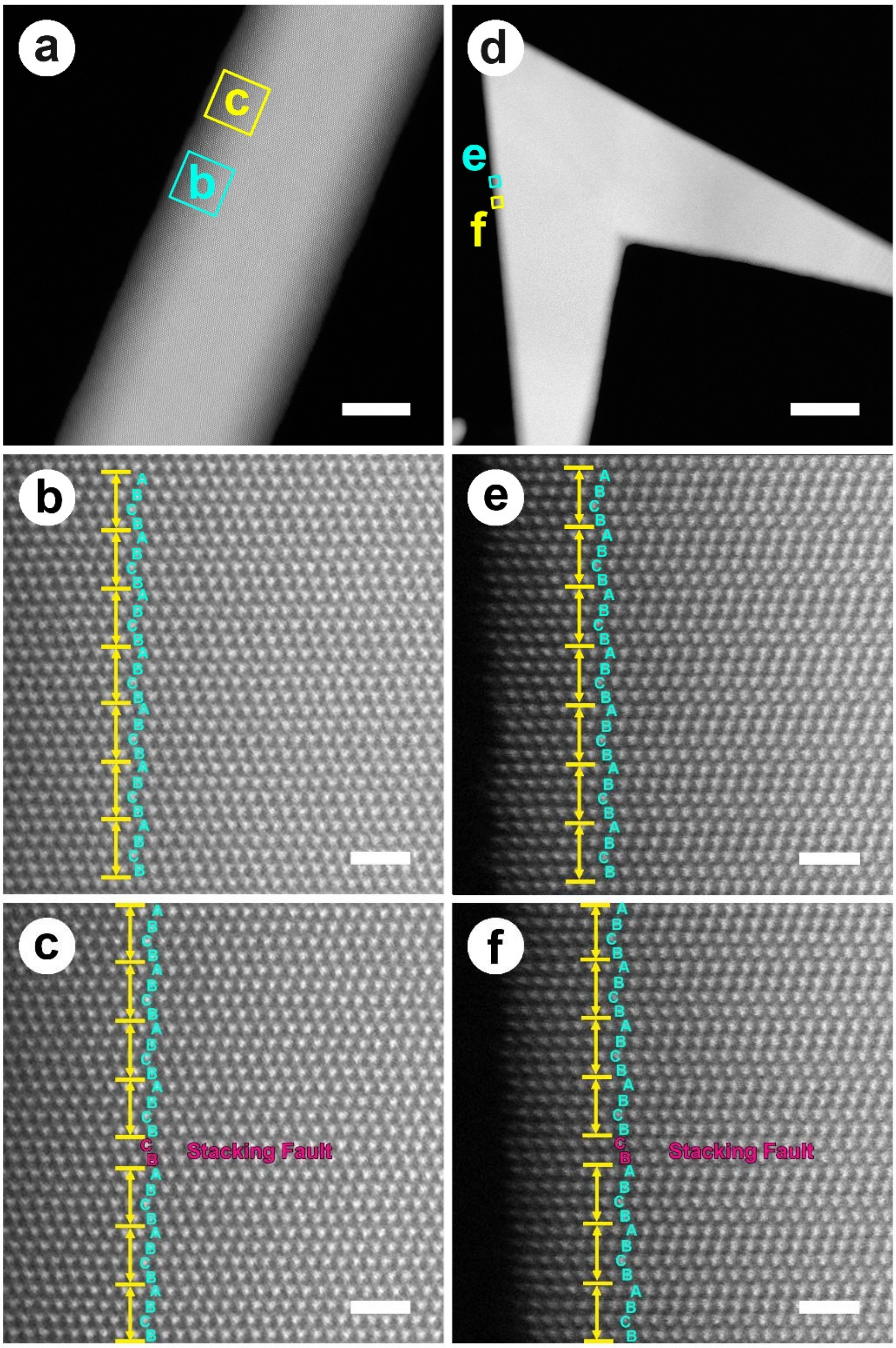

Supplementary Figure 6 | HAADF-STEM images of Au nanoribbons and nanokites. a HAADF-STEM image of an Au nanoribbon. **b** HAADF-STEM image of 4H phase in the turquoise square of the Au nanoribbon in **a**. **c** HAADF-STEM image of stacking faults in the yellow square of the Au nanoribbon in **a**. **d** HAADF-STEM image of an Au nanokite. **e** HAADF-STEM image of 4H phase in the turquoise square of the Au nanokite in **d**. **f** HAADF-STEM image of stacking faults in the yellow square of the Au nanokite in **d**. Scale bars, **a** 10 nm; **b**, **c**, **e**, **f** 1 nm; **d** 50 nm.

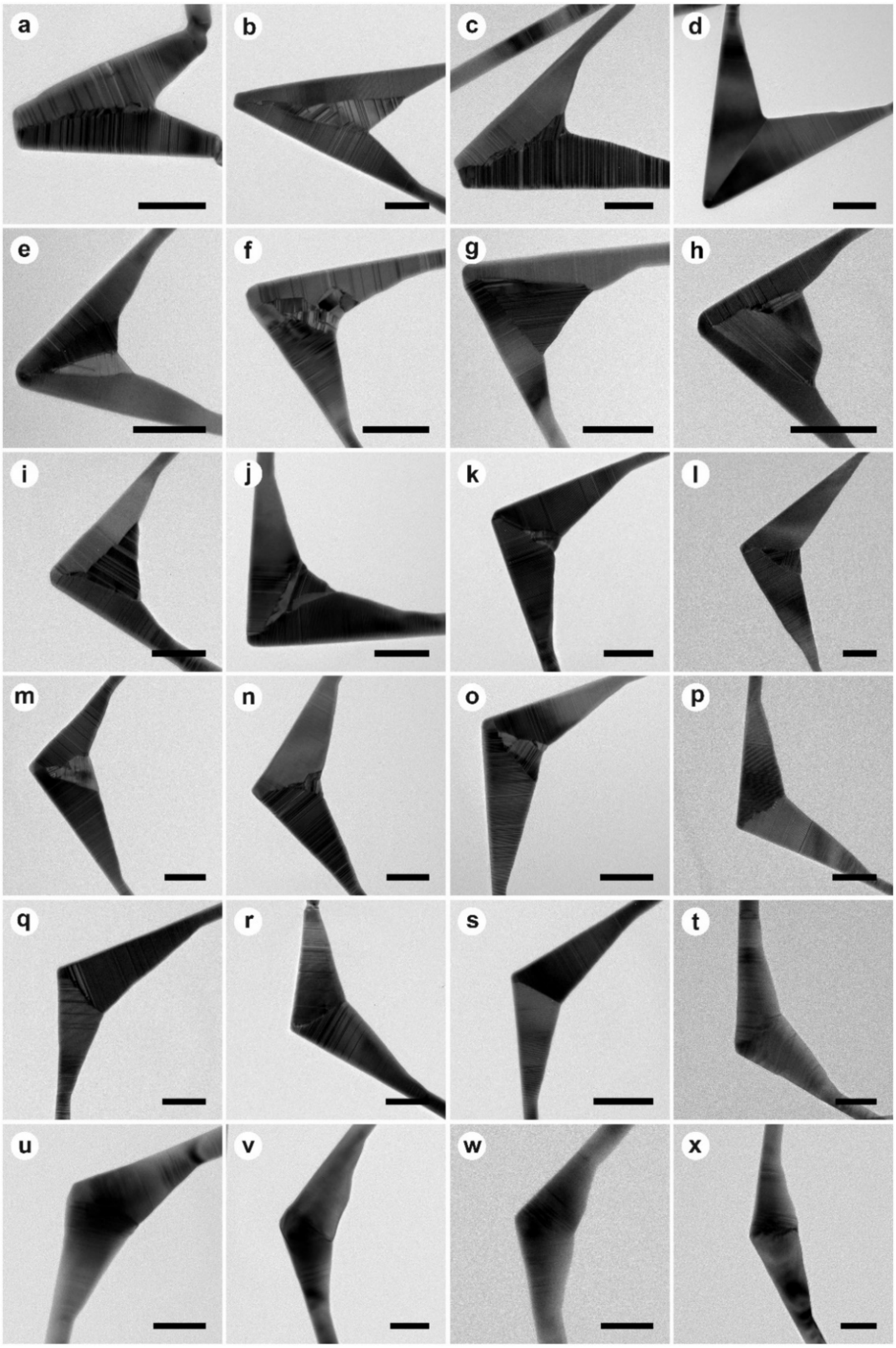

Supplementary Figure 7 | TEM images of the representative Au nanokites with different angles. Except the Au nanokites in **d** and **s** with angles of 54.9º and 115.3º, respectively, possessing well-defined crystal boundaries, the other Au nanokites possess ill-defined crystal boundaries. Scale bars, **a-x** 50 nm.

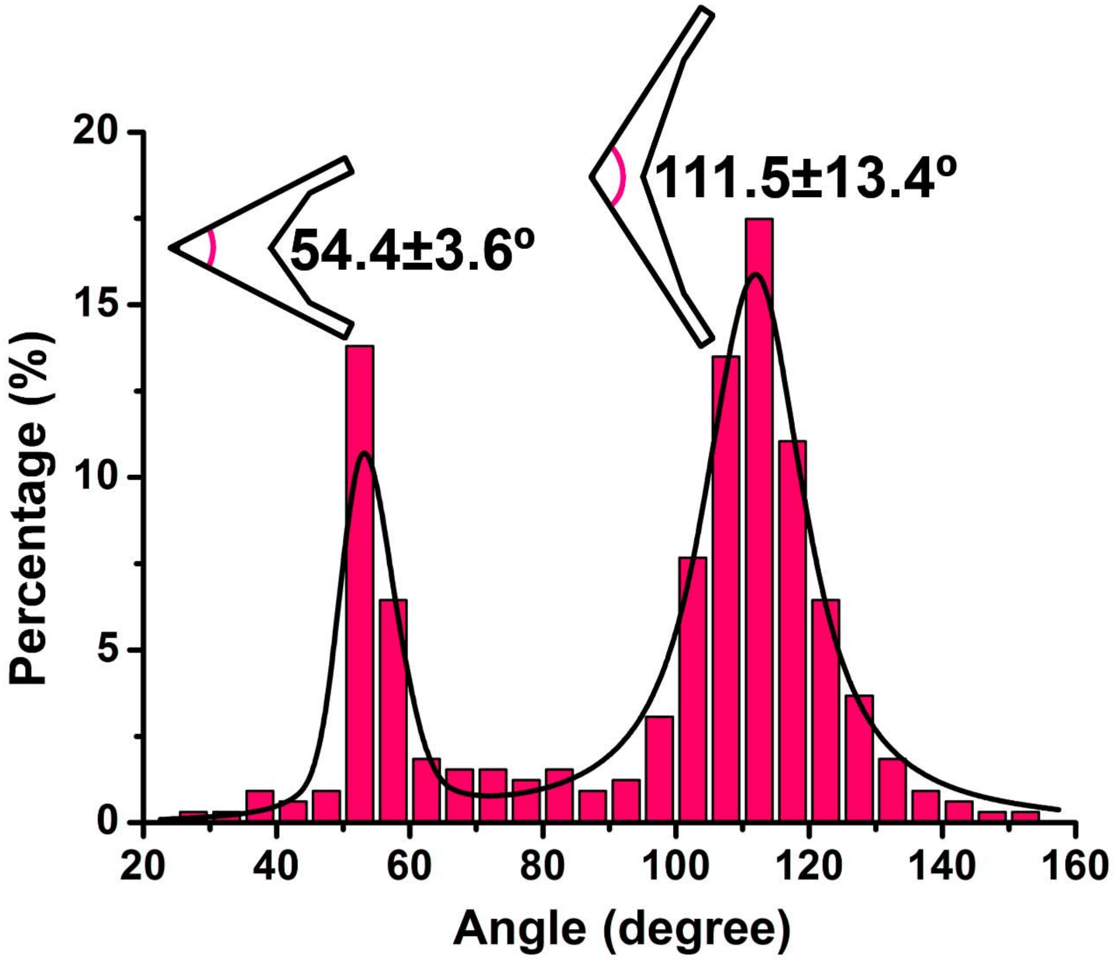

Supplementary Figure 8 | Angle distribution histogram of Au nanokites.

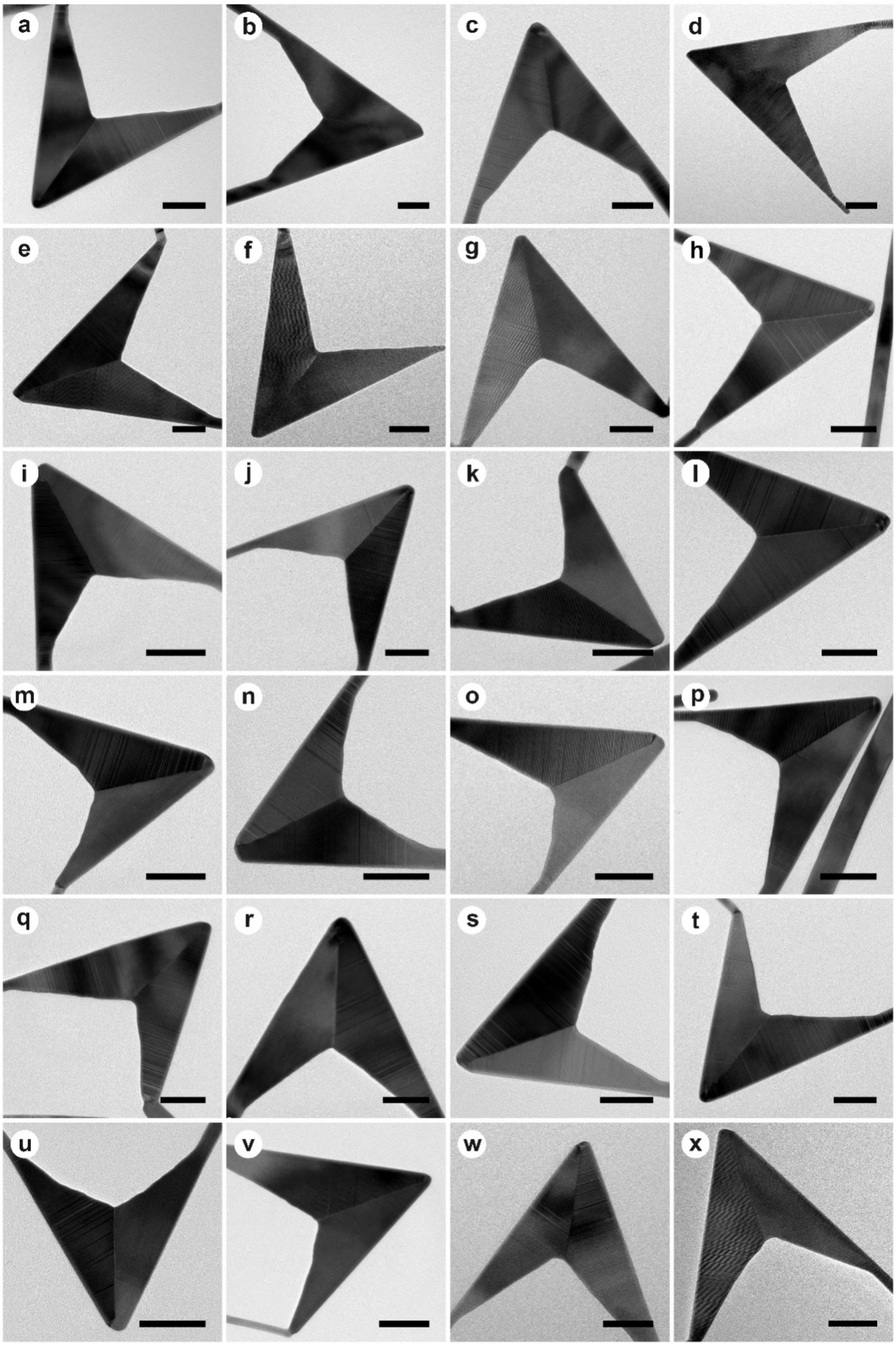

Supplementary Figure 9 | TEM images of the representative twinned Au nanokites with angle of 54.4±3.6º, possessing well-defined crystal boundaries. Scale bars, **a-x** 50 nm.

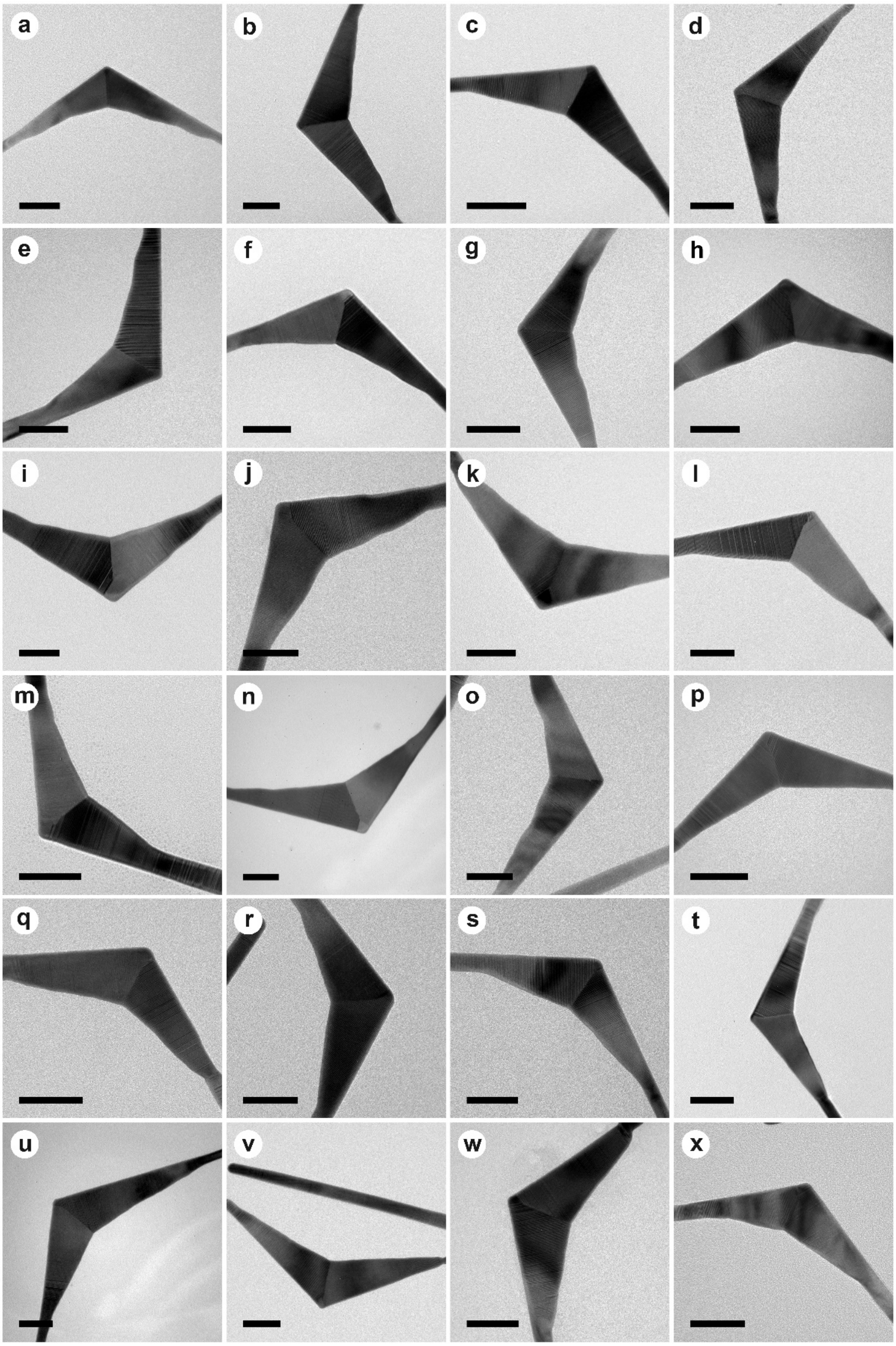

Supplementary Figure 10 | TEM images of representative twinned Au nanokites with angle of 111.5±13.4º, possessing well-defined crystal boundaries. Scale bars, **a**-**x** 50 nm.

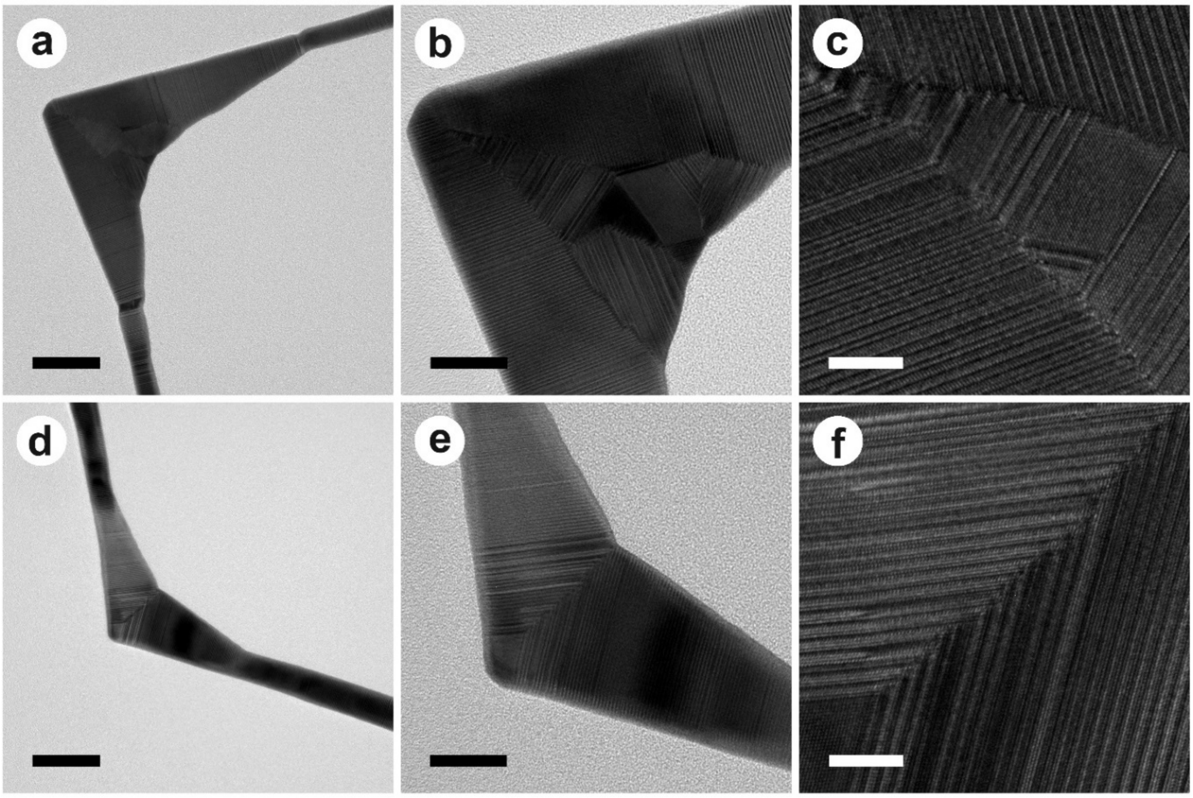

Supplementary Figure 11 | TEM and HRTEM images of two typical Au nanokites with ill-defined crystal boundaries. a-c An acute-angled Au nanokite with ill-defined crystal boundaries. **d-f** An obtuse-angled Au nanokite with ill-defined crystal boundaries. Scale bars, **a, d** 50 nm; **b, e** 20 nm; **c, f** 5 nm.

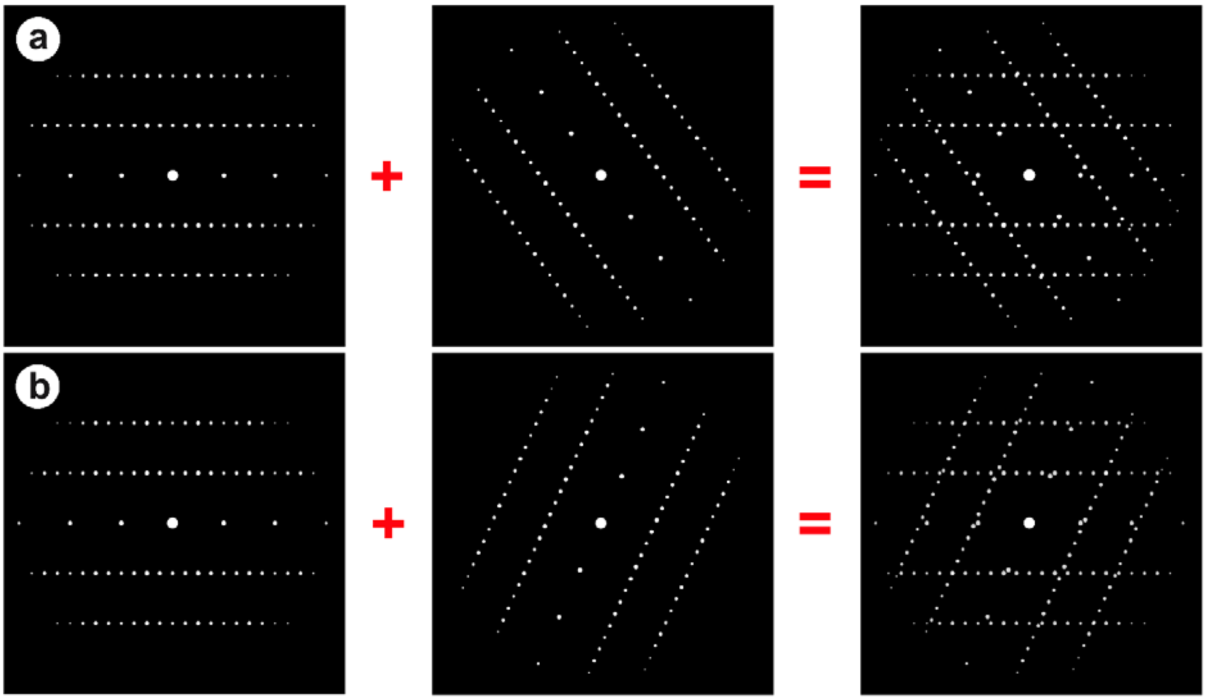

Supplementary Figure 12 | a, b Simulated SAED patterns of the acute-angled a) and obtuse-angled b) twinned Au nanokites. The patterns are generated by superimposing two sets of SAED patterns of 4H-phase sub-crystals along the $[1\bar{2}10]$ direction, with one set rotated by a certain angle with respect to the other. For acute-angled twinned nanokites, the angle of rotation is 54.4º. For obtuse-angled twinned nanokites, the angle of rotation is 114.0º. Note that 54.4º and 114.0º are based on the calculation data shown in Supplementary Table 1. The *fcc* sub-crystal at the tip of arrow-shaped head of the Au nanokite was not considered in the simulation because of its small size and undetectable diffraction (Figs. 2e, 2m).

Supplementary Methods

Calculation of the angles for twinned nanokites with different twinning planes.

An ideal twinned Au nanokite is composed of two pieces of 4H-phase sub-crystals, and these two pieces of 4H-phase sub-crystals are mirror images for each other (Supplementary Figs. 13a and 14a). If the angle of one 4H-phase sub-crystal is designated as θ, the angle of twinned Au nanokite is 2θ**.**

The value of θ from different twinning planes can be calculated from the unit cell parameters of 4H-phase Au. As shown in Supplementary Figs. 13b and 14b, the 4H-phase sub-crystals are aligned with the crystal lattice of 4H-phase Au. Depending on different twinning planes, including $\{10\bar{1}\mathrm{T}\}$ (T=1, 2, 3, 4, 5, 6, 7), θ can be calculated based on the following equation:

$$\theta_{\mathrm{T}} = \tan^{-1}\left(\frac{\mathrm{T}\times\frac{\sqrt{3}}{2}\times a}{c}\right) \qquad (1)$$

where T is the number in twinning planes $\{10\bar{1}\mathrm{T}\}$ (T=1, 2, 3, 4, 5, 6, 7), and *a* (2.866 Å) and *c* (9.662 Å) are the unit cell parameters of 4H-phase Au.

Based on the aforementioned equation, the angles for nanokites with different twinning planes are calculated, which are shown in Supplementary Table 1. The details of calculation of $\{10\bar{1}2\}$ and $\{10\bar{1}6\}$ twinning planes are shown in Supplementary Figs. 13 and 14, respectively.

Calculation of twinning directions of different twinning planes.

The twinning direction of the $\{10\bar{1}\mathrm{T}\}$ twinning plane is perpendicular to two directions, i.e. $<10\bar{1}\mathrm{T}>$ and $<1\bar{2}10>$ (Supplementary Figs. 13 and 14). Suppose that the twinning direction is $<a, b, c, d>$, the scalar product between the $<a, b, c, d>$ and $<10\bar{1}\mathrm{T}>$ (or $<1\bar{2}10>$) must be zero. Therefore, the following equations can be obtained:

$$a \times 1 + b \times 0 + c \times (-1) + d \times \mathrm{T} = 0 \quad (2)$$

$$a \times 1 + b \times (-2) + c \times 1 + d \times 0 = 0 \quad (3)$$

In addition, for the Miller-Bravais indices of hexagonal lattices, the following equation can be obtained:

$$c = -(a + b) \quad (4)$$

Based on the aforementioned three equations, the following correlations are obtained:

$$b = 0 \quad (5)$$

$$c = -a \quad (6)$$

$$d = -\frac{2}{\mathrm{T}} a \quad (7)$$

Therefore, twinning directions of different $\{10\bar{1}\mathrm{T}\}$ twinning planes are calculated to be <$\bar{\mathrm{T}}$0T2> (T=1, 3, 5, 7) or <$\frac{\bar{\mathrm{T}}}{2}$ 0 $\frac{\mathrm{T}}{2}$ 1> (T=2, 4, 6).

Supplementary Table 1 | Calculated angles and twinning directions for twinned Au nanokites with different twinning planes.

Twinning plane	Twinning direction	tan(θ)	θ	2θ
$\{10\bar{1}1\}$	$\langle\bar{1}012\rangle$	0.25688	14.4	28.8
$\{10\bar{1}2\}$	$\langle\bar{1}011\rangle$	0.51376	27.2	54.4
$\{10\bar{1}3\}$	$\langle\bar{3}032\rangle$	0.77063	37.6	75.2
$\{10\bar{1}4\}$	$\langle\bar{2}021\rangle$	1.02751	45.8	91.6
$\{10\bar{1}5\}$	$\langle\bar{5}052\rangle$	1.28439	52.1	104.2
$\{10\bar{1}6\}$	$\langle\bar{3}031\rangle$	1.54127	57.0	114.0
$\{10\bar{1}7\}$	$\langle\bar{7}072\rangle$	1.79815	60.9	121.8

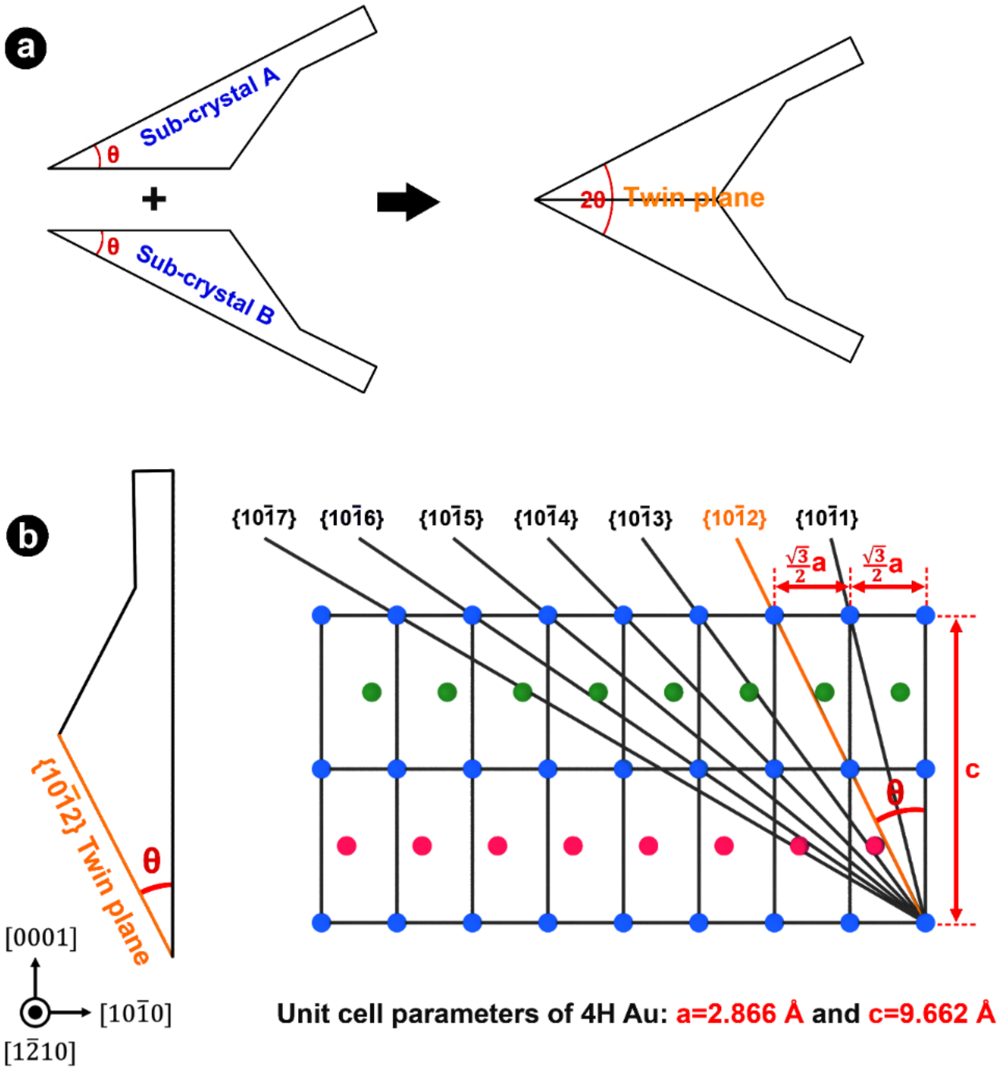

Supplementary Figure 13 | Calculation of the angles for twinned nanokites with {10$\bar{1}$2} twinning plane. a An ideal twinned Au nanokite is composed of two pieces of 4H-phase sub-crystals, i.e. sub-crystal A and sub-crystal B. The angle of one 4H-phase sub-crystal is designated as θ. **b** A 4H-phase sub-crystal aligned with the 4H-phase crystal lattice of Au, and schematic models of different twinning planes of 4H-phase Au lattice viewed in the [1$\bar{2}$10] direction.

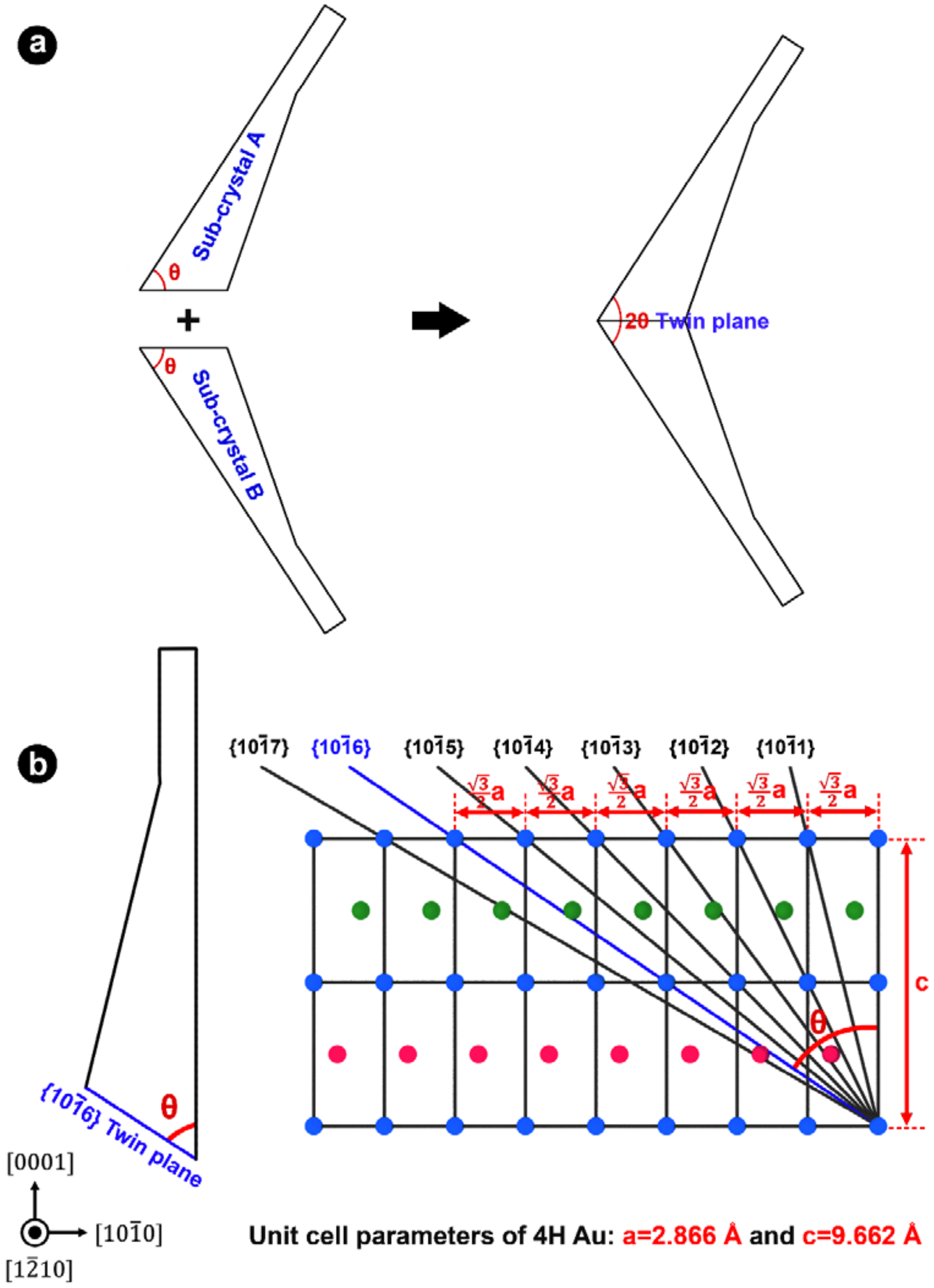

Supplementary Figure 14 | Calculation of the angles for twinned nanokites with {10$\bar{1}$6} twinning plane. a An ideal twinned Au nanokite is composed of two pieces of 4H-phase sub-crystals, i.e. sub-crystal A and sub-crystal B. The angle of one 4H-phase sub-crystal is designated as θ. **b** A 4H-phase sub-crystal aligned with the 4H-phase crystal lattice of Au, and schematic models of different twinning planes of 4H-phase Au lattice viewed in the [1$\bar{2}$10] direction.

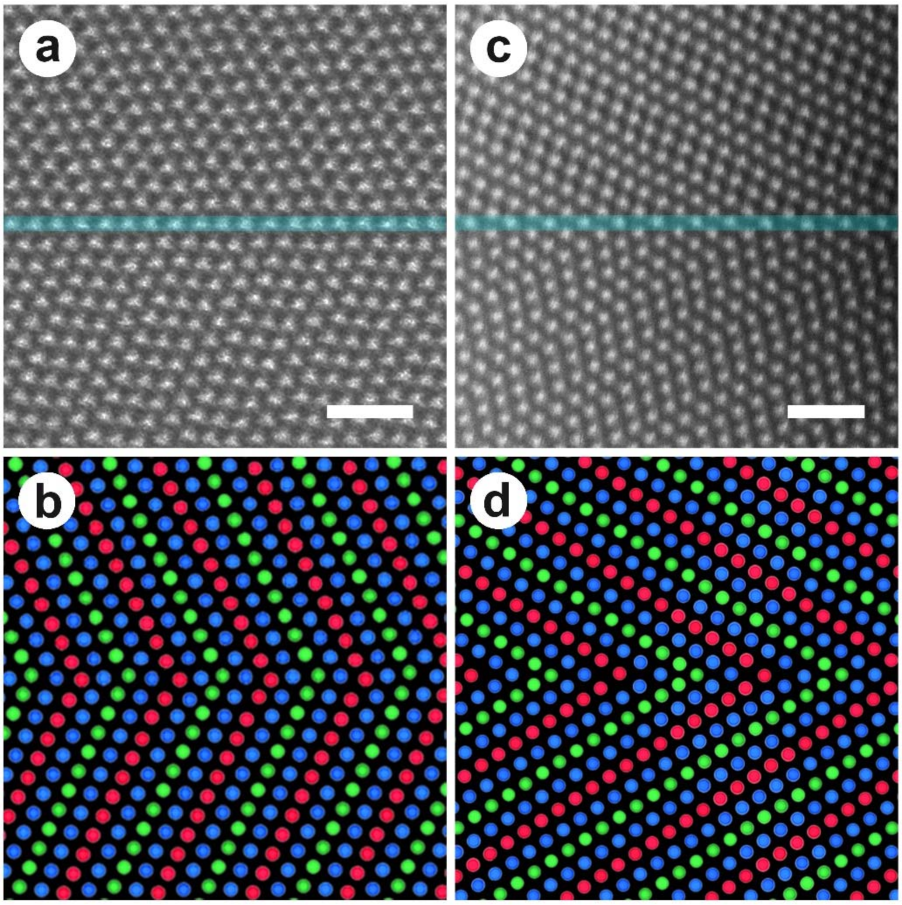

Supplementary Figure 15 | HAADF-STEM images and atomic models of twin boundaries of Au nanokites. a, b HAADF-STEM image and atomic model of the twin boundary of acute Au nanokite. **c, d** HAADF-STEM image and atomic model of the twin boundary of obtuse Au nanokite. The twin boundaries are marked in turquoise lines. Scale bars, **a, c** 1 nm.

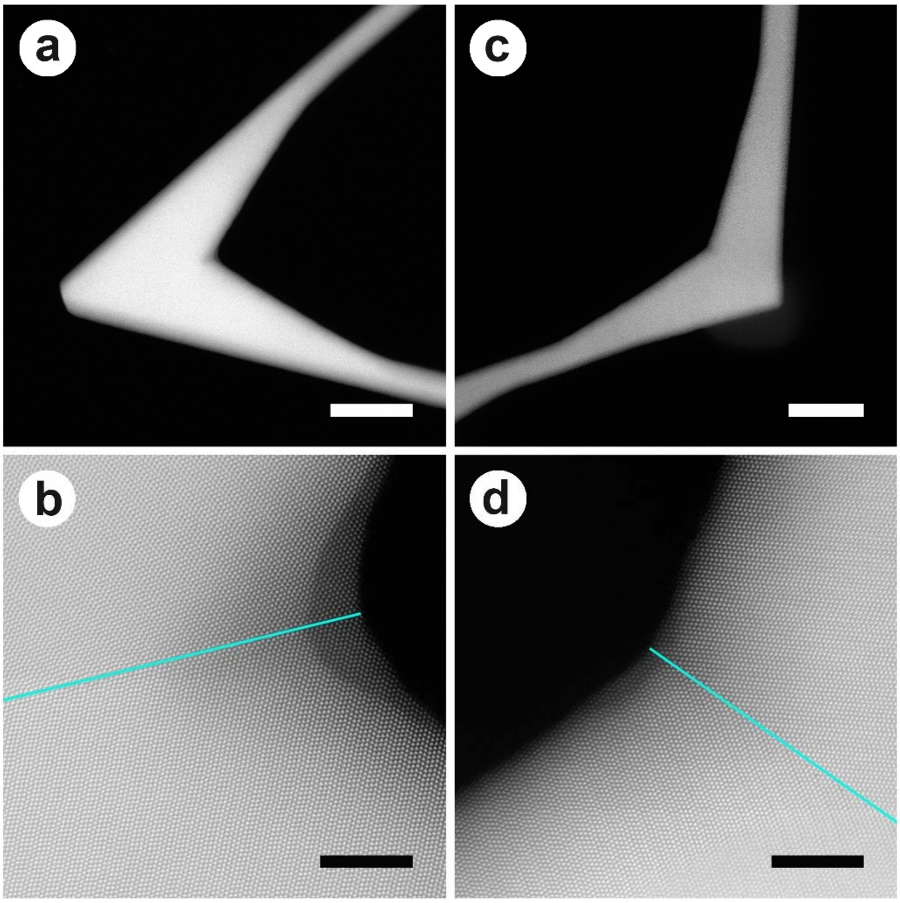

Supplementary Figure 16 | HAADF-STEM images of acute and obtuse Au@Ag nanokites. a HAADF-STEM image of an acute-angled Au@Ag nanokite. **b** Atomic HAADF-STEM image of the acute-angled Au@Ag nanokite in **a**. **c** HAADF-STEM image of an obtuse-angled Au@Ag nanokite. **d** Atomic HAADF-STEM image of the obtuse-angled Au@Ag nanokite in **c**. The twin boundaries are marked in turquoise lines. Scale bars, **a, c** 50 nm; **b, d** 5 nm.

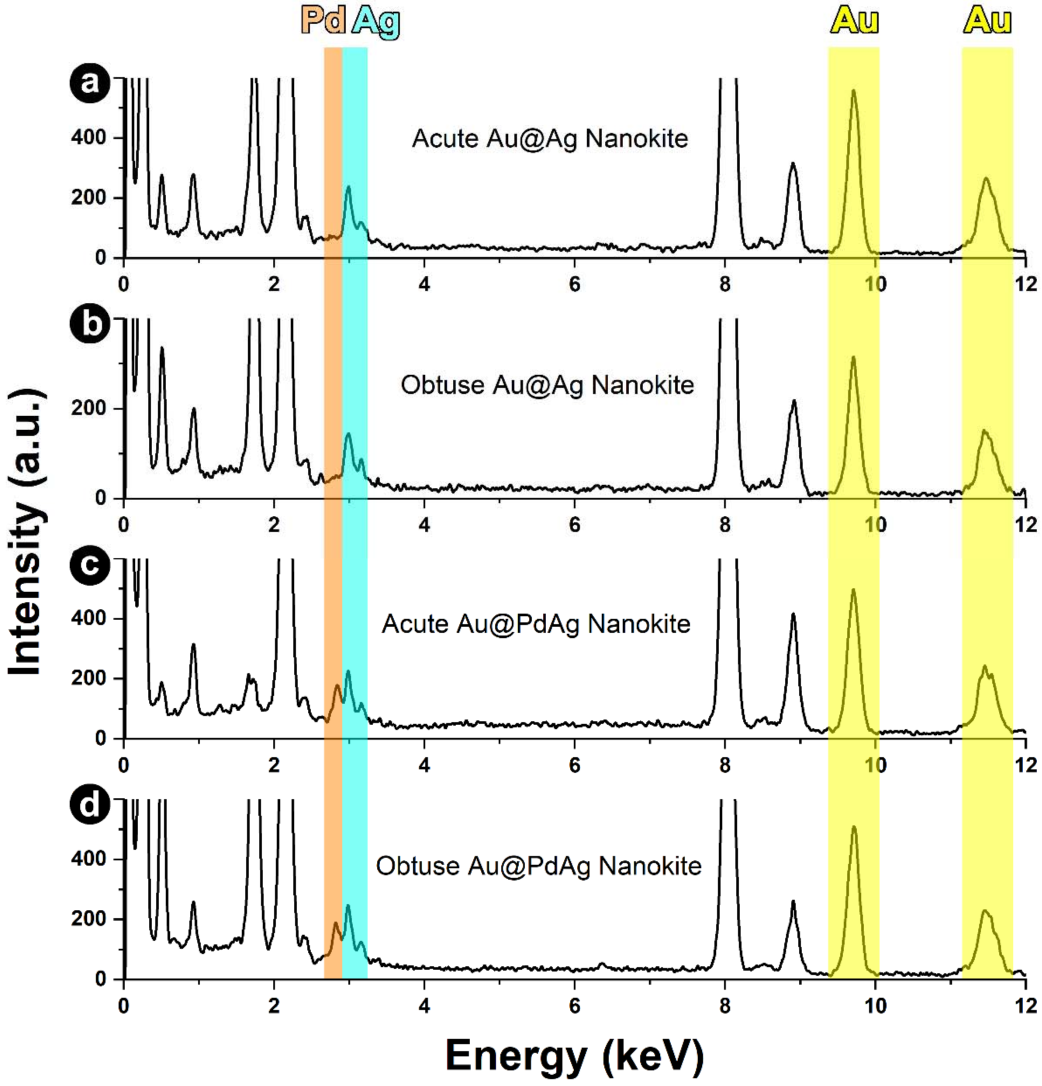

Supplementary Figure 17 | Representative EDX spectra of multimetallic nanokites. **a** Acute-angled Au@Ag nanokite sample (Au/Ag atomic ratio of 1.00/0.30). **b** Obtuse-angled Au@Ag nanokite sample (Au/Ag atomic ratio of 1.00/0.37). **c** Acute-angled Au@PdAg nanokite sample (Au/Ag/Pd atomic ratio of 1.00/0.14/0.14). **d** Obtuse-angled Au@PdAg nanokite sample (Au/Ag/Pd atomic ratio of 1.00/0.20/0.19).

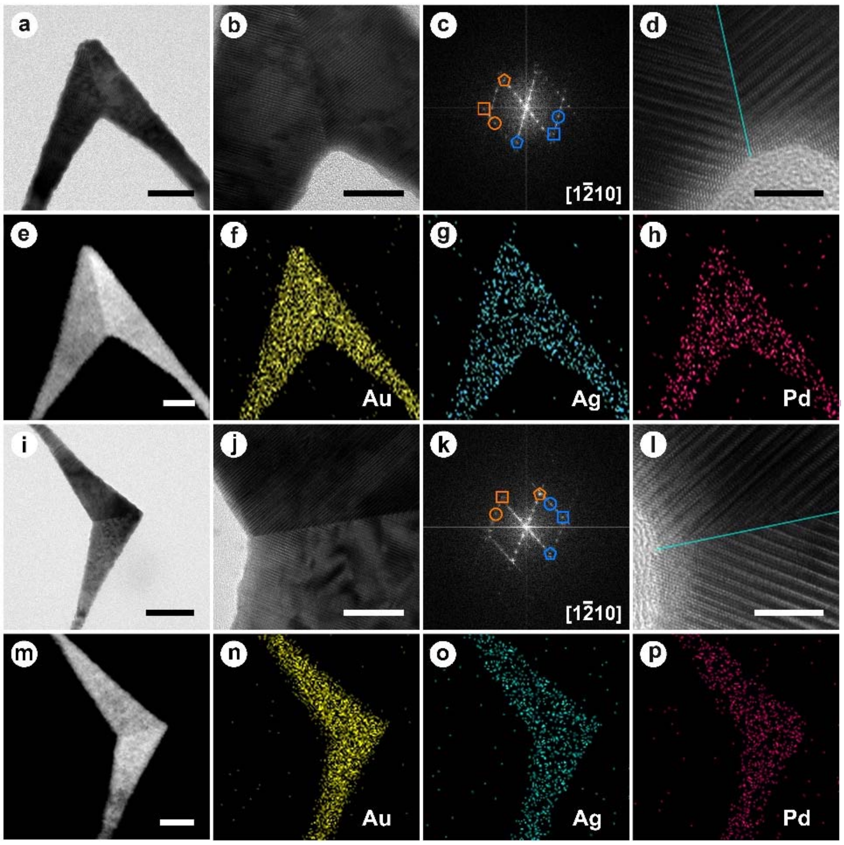

Supplementary Figure 18 | Characterizations of Au@PdAg twinned nanokites. a TEM image of an acute-angled Au@PdAg nanokite. **b, c** High-magnification TEM image and corresponding FFT pattern of the acute-angled Au@PdAg nanokite. (0004), (10$\bar{1}$0), and (10$\bar{1}$2) spots of 4H phase are marked with pentagons, circles, and squares, respectively. The orange and blue represent different 4H sub-crystals. **d** HRTEM image of the twin boundary of the acute-angled Au@PdAg twinned nanokite. The twin boundary is marked in turquoise line. **e** STEM and **f-h** STEM-EDX elemental mapping images of an acute-angled Au@PdAg nanokite. **i** TEM image of an obtuse-angled Au@PdAg nanokite. **j, k** High-magnification TEM image and corresponding FFT pattern of the obtuse-angled Au@PdAg nanokite. **l** HRTEM image of the twin boundary of the obtuse-angled Au@PdAg twinned nanokite. The twin boundary is marked in turquoise line. **m** STEM and **n-p** STEM-EDX elemental mapping images of an obtuse-angled Au@PdAg nanokite. Scale bars, **a, e, m**, 50 nm; **b, j**, 20 nm; **d, l**, 5 nm; **i**, 100 nm.

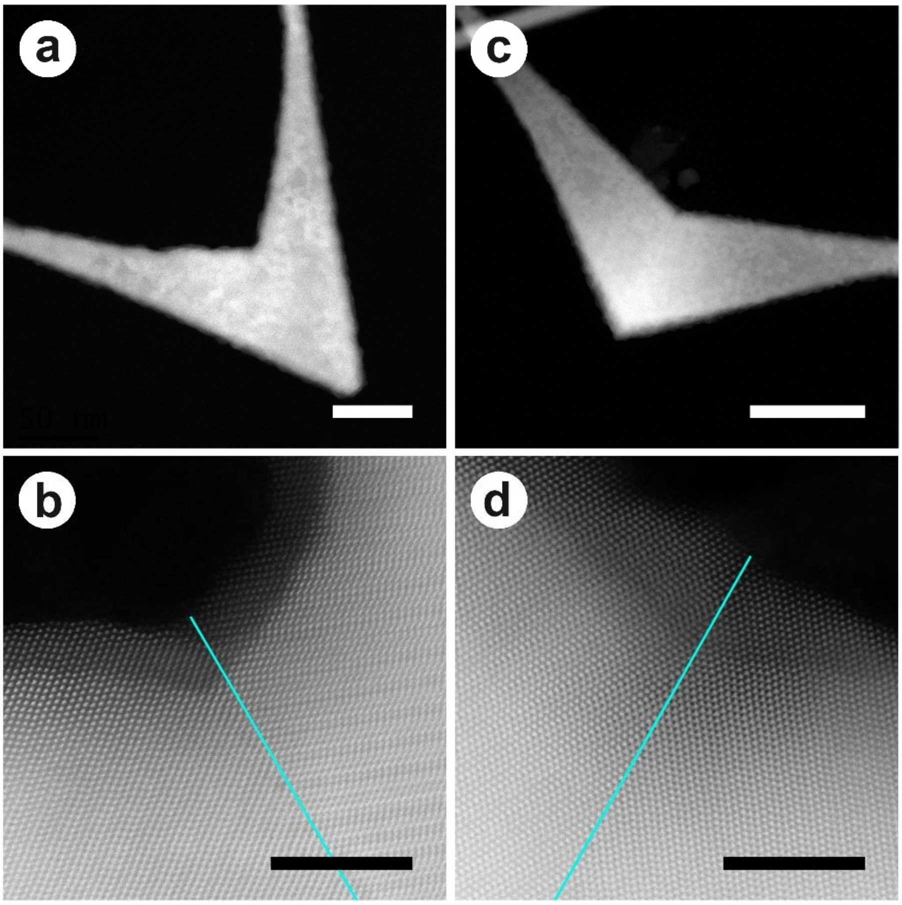

Supplementary Figure 19 | HAADF-STEM images of acute and obtuse Au@PdAg nanokites. **a** HAADF-STEM image of an acute-angled Au@PdAg nanokite. **b** Atomic HAADF-STEM image of the acute-angled Au@PdAg nanokite in **a**. **c** HAADF-STEM image of an obtuse-angled Au@PdAg nanokite. **d** Atomic HAADF-STEM image of the obtuse-angled Au@PdAg nanokite in **c**. The twin boundaries are marked in turquoise lines. Scale bars, **a** 50 nm; **b, d** 5 nm; **c** 100 nm.
